# Supplementary material for: Association between deep learning–based atrial fibrillation burden and in-hospital mortality
Source: PLOS Digit Health. 2026 Mar 4;5(3):e0001266. doi: 10.1371/journal.pdig.0001266 (PMC12959658; doi:10.1371/journal.pdig.0001266)
Supplement: S1 Method — (DOCX) [file pdig.0001266.s001.docx]

**S1 Method: PTB-XL data**

The PTB-XL dataset[1] was collected using devices from Schiller AG between October 1989 and June 1996. The dataset comprises 21,837 clinical 12-lead electrocardiogram (ECG) records, each 10 s in length, from 18,885 patients. The ECG records were annotated by up to two cardiologists with potentially multiple ECG statements from a set of 71 different statements conforming to the SCP-ECG standard. As the waveform in the Medical Information Mart for Intensive Care III (MIMIC-III) database was 125 Hz, the PTB-XL ECG waveform was resampled to 125 Hz using the scipy.signal.resample function.

1. Wagner P, Strodthoff N, Bousseljot RD, Kreiseler D, Lunze FI, Samek W, et al. PTB-XL, a large publicly available electrocardiography dataset. Sci Data. 2020;7(1):154. Epub 20200525. doi: 10.1038/s41597-020-0495-6. PubMed PMID: 32451379; PubMed Central PMCID: PMCPMC7248071.
