# Supplementary material for: Association between deep learning–based atrial fibrillation burden and in-hospital mortality
Source: PLOS Digit Health. 2026 Mar 4;5(3):e0001266. doi: 10.1371/journal.pdig.0001266 (PMC12959658; doi:10.1371/journal.pdig.0001266)
Supplement: S2 Method — (DOCX) [file pdig.0001266.s002.docx]

**S2 Method: AF 2017 Challenge data**

The AF 2017 challenge dataset[1] contains ECG recordings collected using the AliveCor device. These ECG recordings were generously donated to Challenge by AliveCor. It comprises 12,186 single-lead ECG recordings collected using AliveCor devices. The dataset includes four classes of ECG recordings: normal rhythm, atrial fibrillation (AF), other abnormal rhythms, and noisy recordings. Labelling was refined during the challenge to address initial noise and misclassifications, thereby improving accuracy.

1. Clifford GD, Liu C, Moody B, Lehman LH, Silva I, Li Q, et al. AF Classification from a Short Single Lead ECG Recording: the PhysioNet/Computing in Cardiology Challenge 2017. Comput Cardiol (2010). 2017;44. Epub 20180405. doi: 10.22489/CinC.2017.065-469. PubMed PMID: 29862307; PubMed Central PMCID: PMCPMC5978770.
