# Supplementary material for: Association between deep learning–based atrial fibrillation burden and in-hospital mortality
Source: PLOS Digit Health. 2026 Mar 4;5(3):e0001266. doi: 10.1371/journal.pdig.0001266 (PMC12959658; doi:10.1371/journal.pdig.0001266)
Supplement: S3 Method — (DOCX) [file pdig.0001266.s003.docx]

**S3 Method: Shaoxing Hospital data**

The Shaoxing Hospital database[1] is a 12-lead ECG database for arrhythmia research collected from Shaoxing People’s Hospital (Shaoxing Hospital Zhejiang University School of Medicine). This database was created for arrhythmia research and contains 12-lead ECG signals from 10,646 patients. Each patient’s ECG was recorded for 10 s at a sampling rate of 500 Hz. The dataset includes expert-labelled information on 11 common rhythms and 67 additional cardiovascular conditions.

1. Zheng J, Zhang J, Danioko S, Yao H, Guo H, Rakovski C. A 12-lead electrocardiogram database for arrhythmia research covering more than 10,000 patients. Scientific data. 2020;7(1):48.
