# Supplementary material for: Association between deep learning–based atrial fibrillation burden and in-hospital mortality
Source: PLOS Digit Health. 2026 Mar 4;5(3):e0001266. doi: 10.1371/journal.pdig.0001266 (PMC12959658; doi:10.1371/journal.pdig.0001266)
Supplement: S4 Method — (DOCX) [file pdig.0001266.s004.docx]

**S4 Method: Lobachevsky University Electrocardiography Database (LUDB)**

The Lobachevsky University Electrocardiography Database (LUDB)[1] is an ECG signal database with marked boundaries and peaks of P and T waves and QRS complexes. The database consists of 200 10-s 12-lead ECG signal records representing different morphologies of the ECG signal. ECGs were collected from healthy volunteers and patients at Nizhny Novgorod City Hospital No. 5 between 2017 and 2018. The patients had various cardiovascular diseases, and some had pacemakers. The age of all volunteers ranged from a minimum of 11 years to a maximum of >89 years, with an average age of 52 years. The distribution by sex was 85 women and 115 men.

1. Kalyakulina AI, Yusipov II, Moskalenko VA, Nikolskiy AV, Kosonogov KA, Osipov GV, et al. LUDB: a new open-access validation tool for electrocardiogram delineation algorithms. IEEE access. 2020;8:186181-90.
