# Supplementary material for: Association between deep learning–based atrial fibrillation burden and in-hospital mortality
Source: PLOS Digit Health. 2026 Mar 4;5(3):e0001266. doi: 10.1371/journal.pdig.0001266 (PMC12959658; doi:10.1371/journal.pdig.0001266)
Supplement: S5 Method — (DOCX) [file pdig.0001266.s005.docx]

**S5 Method: MIT-BIH Arrhythmia database**

The MIT-BIH Arrhythmia Database[1] was developed by researchers at the Beth Israel Hospital and MIT between 1975 and 1979. The database contains 48 half-hour excerpts of two-channel ECG recordings from 47 patients, of which 23 were selected randomly, and the other 25 were chosen for containing uncommon but clinically important arrhythmias. This dataset is instrumental in the advancement of automated arrhythmia-detection algorithms and has been widely used in both academic research and industry, thereby influencing the development of medical devices and evaluation techniques. This remains a key reference point for cardiac signal analysis.

1. Moody GB, Mark RG. The impact of the MIT-BIH arrhythmia database. IEEE engineering in medicine and biology magazine. 2001;20(3):45-50.
