# Supplementary material for: Association between deep learning–based atrial fibrillation burden and in-hospital mortality
Source: PLOS Digit Health. 2026 Mar 4;5(3):e0001266. doi: 10.1371/journal.pdig.0001266 (PMC12959658; doi:10.1371/journal.pdig.0001266)
Supplement: S6 Method — (DOCX) [file pdig.0001266.s006.docx]

**S6 Method: Deep-learning model for rhythm classification**

A deep learning model was trained to classify AF and pacemaker rhythms from MIMIC-III ECG waveforms. Four ResNet models were trained with ECG waveforms from the PTB-XL and AF Challenge 2017 datasets. The dataset was split in a ratio of 6:2:2. The models were externally validated using the ECG waveforms from the Shaoxing Hospital ECG dataset (Table S2). Four models were used: ResNet-18, ResNet-34, SE-ResNet-18, and SE-ResNet-34, and each model was configured to process one-dimensional data by setting the size of the input data to (1250, 1). The batch size was set to 16, and the learning rate was set to vary periodically over a small range using a Cyclical Learning Rate. The minimum learning rate was set to 1e-6, and the maximum learning rate was set to 1e-3. The optimizer was set to Adam, and the ModelCheckpoint callback was set to save the best-performing model. The best-performing model was selected as that with the lowest validation loss. A binary cross-entropy loss function and accuracy metric were used to construct the models. Finally, each model was trained for 100 epochs using 16 samples from each batch, and the data were shuffled. The best-performing models were saved, and validation data were used to evaluate model performance during training.

In addition, SE-ResNet-34, which previously performed best in AF rhythm classification, was trained to classify pacemaker rhythms for exclusion. The PTB-XL and LUDB databases were used for model training. The dataset was split in a ratio of 8:2. Model performance was evaluated using the MIT-BIH Arrhythmia database (Table S3). The Cyclical Learning Rate was set to vary the learning rate periodically, starting with an initial learning rate of 1e-6 and increasing to a maximum of 1e-3. The optimizer was set to Adam, and the binary cross-entropy loss function, which is suitable for binary classification, was used. During model training, a ModelCheckpoint callback was used to save only the best-performing weights. Training was performed for 100 epochs, and the data were divided into batch sizes of 16.
