# Supplementary material for: Association between deep learning–based atrial fibrillation burden and in-hospital mortality
Source: PLOS Digit Health. 2026 Mar 4;5(3):e0001266. doi: 10.1371/journal.pdig.0001266 (PMC12959658; doi:10.1371/journal.pdig.0001266)
Supplement: S1 Table — (DOCX) [file pdig.0001266.s007.docx]

**S1 Table. Performance of in-hospital mortality prediction models in critically ill patients**

| Threshold = 0.5 |  | **Logistic regression** | **Random forest** | **XG boost** |
| --- | --- | --- | --- | --- |
| Accuracy |  | 0.767 | 0.791 | 0.579 |
| Sensitivity |  | 0.792 | 0.804 | 0.173 |
| Specificity |  | 0.743 | 0.778 | 0.988 |
| F1 score |  | 0.774 | 0.794 | 0.293 |
| AUROC |  | 0.86 | 0.86 | 0.80 |

Atrial fibrillation burden was used for modelling in addition to other clinical variables.

AUROC, area under receiver operating characteristic curve.
