# Supplementary material for: Association between deep learning–based atrial fibrillation burden and in-hospital mortality
Source: PLOS Digit Health. 2026 Mar 4;5(3):e0001266. doi: 10.1371/journal.pdig.0001266 (PMC12959658; doi:10.1371/journal.pdig.0001266)
Supplement: S2 Table — (DOCX) [file pdig.0001266.s008.docx]

**S2 Table. Performance of the atrial fibrillation classification model using external validation dataset**

| Threshold = 0.5 |  | **ResNet-18** | **ResNet-34** | **SE-ResNet-18** | **SE-ResNet-34** |
| --- | --- | --- | --- | --- | --- |
| Accuracy |  | 0.875 | 0.931 | 0.881 | 0.943 |
| Sensitivity |  | 0.971 | 0.952 | 0.963 | 0.937 |
| Specificity |  | 0.850 | 0.925 | 0.860 | 0.945 |
| PPV |  | 0.630 | 0.771 | 0.644 | 0.817 |
| F1 score |  | 0.765 | 0.852 | 0.772 | 0.873 |
| AUROC |  | 0.959 | 0.984 | 0.956 | 0.983 |

PPV, positive predictive value; AUROC, area under receiver operating characteristic curve.
