# Supplementary material for: Association between deep learning–based atrial fibrillation burden and in-hospital mortality
Source: PLOS Digit Health. 2026 Mar 4;5(3):e0001266. doi: 10.1371/journal.pdig.0001266 (PMC12959658; doi:10.1371/journal.pdig.0001266)
Supplement: S3 Table — (DOCX) [file pdig.0001266.s009.docx]

**S3 Table. Performance of the pacemaker rhythm classification model**

| Threshold = 0.5 |  | **SE-ResNet-34** |
| --- | --- | --- |
| Accuracy |  | 0.985 |
| Sensitivity |  | 0.919 |
| Specificity |  | 0.991 |
| PPV |  | 0.914 |
| F1 score |  | 0.917 |
| AUROC |  | 0.990 |

PPV, positive predictive value; AUROC, area under receiver operating characteristic curve.
