# Supplementary material for: Association between deep learning–based atrial fibrillation burden and in-hospital mortality
Source: PLOS Digit Health. 2026 Mar 4;5(3):e0001266. doi: 10.1371/journal.pdig.0001266 (PMC12959658; doi:10.1371/journal.pdig.0001266)
Supplement: S4 Table — (DOCX) [file pdig.0001266.s010.docx]

**S4 Table. Feature name and clinical meaning**

| **Feature name** | **Clinical meaning** |
| --- | --- |
| Male Sex | Biological sex (Male) |
| Age | The standard count of time since birth. |
| AF burden | Atrial Fibrillation Burden. The percentage of time the heart spends in Atrial Fibrillation (AF) out of the total recording time. Reflects the severity of AF and atrial functional impairment. |
| Diabetes mellitus | A chronic metabolic disorder characterized by high blood glucose levels. Increases the risk of cardiovascular disease, kidney failure, and infections. |
| Congestive heart failure | A state where the heart cannot pump blood effectively. Often used to denote an acute exacerbation or severe cardiac function deterioration. |
| Chronic liver disease | Long-standing liver dysfunction affecting metabolism, coagulation, immune regulation, and detoxification. |
| Chronic kidney disease | Progressive decline in kidney function over more than three months. Affects fluid balance, electrolytes, and toxin clearance. |
| Chronic pulmonary disease | Long-term conditions (e.g., Chronic Obstructive Pulmonary Disease) that cause progressive reduction in respiratory function. Limits lung capacity and increases susceptibility to infection. |
| Cerebrovascular disease | Disorders of cerebral blood vessels that can cause ischemic or hemorrhagic brain injury and long-term neurologic deficits. (e.g., Stroke, Transient Ischemic Attack). |
| Cancer | Malignant disease associated with systemic inflammation, immune dysregulation, and increased risk of organ failure |
| Connective tissue disease | Autoimmune diseases (e.g., Lupus, Rheumatoid Arthritis) that cause systemic inflammation. |
| Dementia | Chronic, progressive decline in cognitive function. |
| Emergency department | Admission via the emergency department. |
| SOFA score | **Sequential Organ Failure Assessment score.** A scoring system that assesses the severity of acute organ dysfunction across six organ systems: Respiratory, Coagulation, Liver, Cardiovascular, Central Nervous System, and Renal. |
| Renal replacement therapy | **A set of medical treatments that replace the normal blood-filtering function of the kidneys** when they can no longer do so adequately |
| Ventilator use | Indicator of whether the patient required mechanical ventilation, reflecting the presence of severe respiratory failure or critical illness |
| Sepsis | Life-threatening organ dysfunction caused by a dysregulated host response to infection. |
