# Supplementary material for: Association between deep learning–based atrial fibrillation burden and in-hospital mortality
Source: PLOS Digit Health. 2026 Mar 4;5(3):e0001266. doi: 10.1371/journal.pdig.0001266 (PMC12959658; doi:10.1371/journal.pdig.0001266)
Supplement: S1 Fig — A) MIMIC-III training dataset, B) Yongin Severance Hospital external validation dataset. (DOCX) [file pdig.0001266.s011.docx]

**S1 Fig.** **Risk of in-hospital mortality by AF burden.** A) MIMIC-III dataset, B) Yongin Severance Hospital (External Validation)

**A)**

**
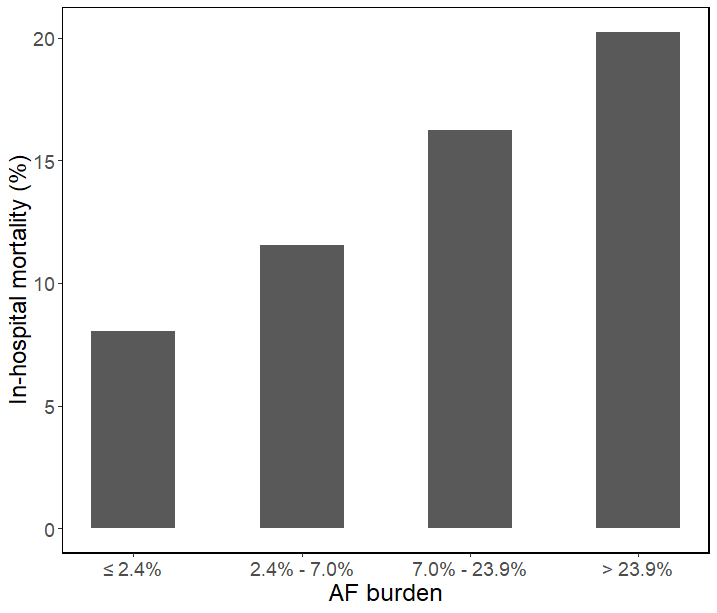
**

**B)**


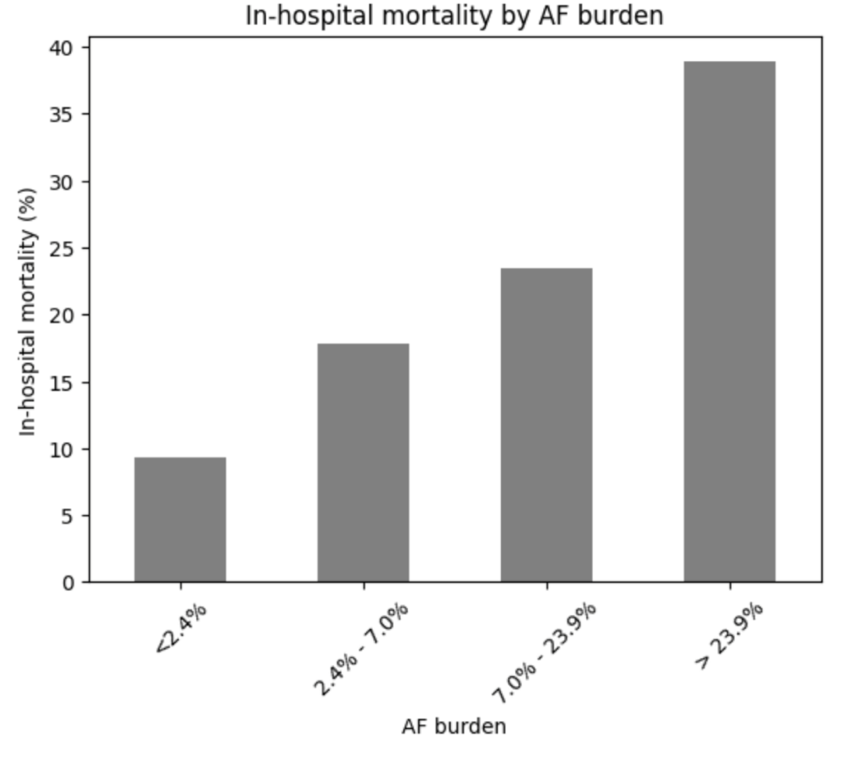


MIMIC-III, Medical Information Mart for Intensive Care III; AF, atrial fibrillation
