# Supplementary material for: Association between deep learning–based atrial fibrillation burden and in-hospital mortality
Source: PLOS Digit Health. 2026 Mar 4;5(3):e0001266. doi: 10.1371/journal.pdig.0001266 (PMC12959658; doi:10.1371/journal.pdig.0001266)
Supplement: S2 Fig — (DOCX) [file pdig.0001266.s012.docx]

**S2 Fig. Restricted cubic spline showing the relationship between AF burden and in-hospital mortality in the MIMIC-III dataset**


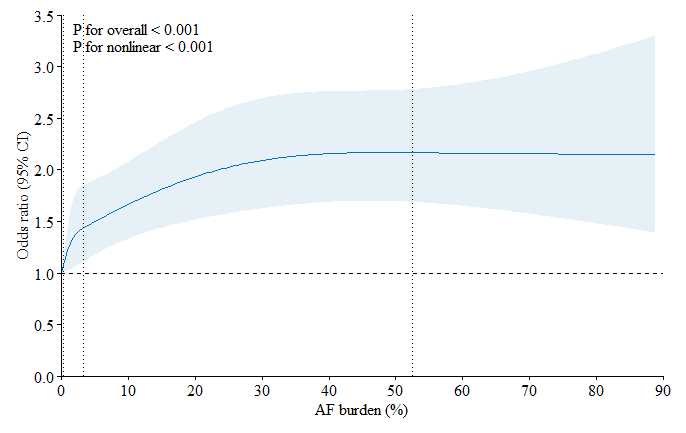


Data were fitted using a logistic regression model with four knots placed at the 5th, 35th, 65th, and 95th percentiles of AF burden (reference = 5th percentile). Covariates that remained significant in the multivariable logistic regression model (Table 3) were included in the model to adjust for confounding.

CI, confidence interval; AF, atrial fibrillation.
