# Supplementary figures and images for: Association between deep learning–based atrial fibrillation burden and in-hospital mortality
Source: PLOS Digit Health. 2026 Mar 4;5(3):e0001266. doi: 10.1371/journal.pdig.0001266 (PMC12959658; doi:10.1371/journal.pdig.0001266)

**S3 Fig. Feature Importance A) MIMIC-III dataset, B) Yongin Severance Hospital**

**A)**


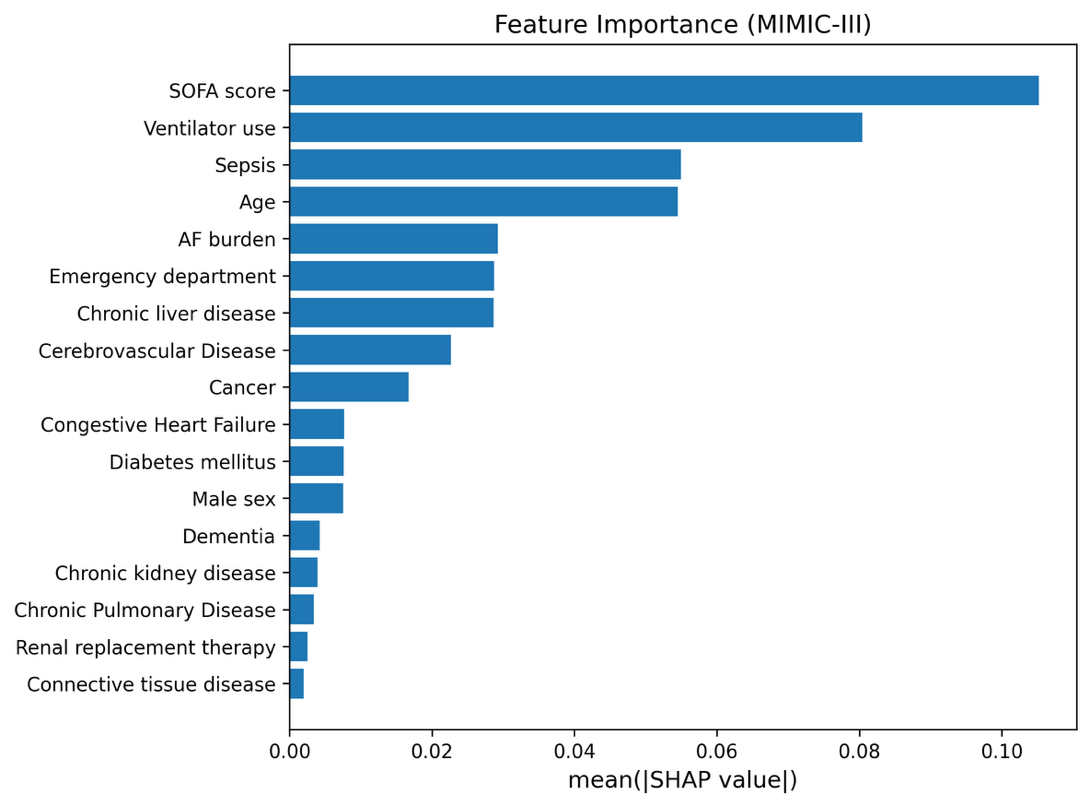


**B)**


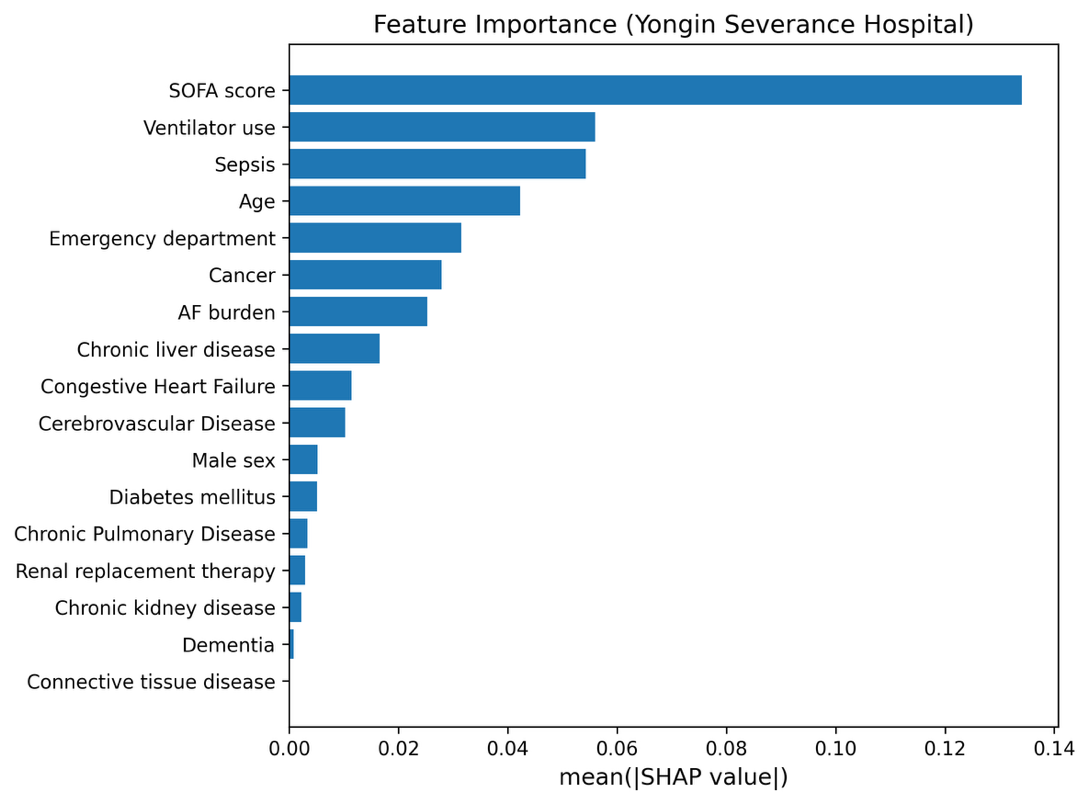

Supplement: S3 Fig — (DOCX) [file pdig.0001266.s013.docx]
