# Supplementary material for: Association between deep learning–based atrial fibrillation burden and in-hospital mortality
Source: PLOS Digit Health. 2026 Mar 4;5(3):e0001266. doi: 10.1371/journal.pdig.0001266 (PMC12959658; doi:10.1371/journal.pdig.0001266)
Supplement: Attachment — Submitted filename: Response to Reviewers.docx [file pdig.0001266.s015.docx]

**Dear Editor and Reviewers,**
We greatly appreciate the detailed and constructive feedback on our manuscript. Your comments have provided valuable guidance and have enabled us to refine the study further. We have addressed all points in the revised version and hope that the improvements meet your expectations.

Editor’s comment

1. **Please include a table describing the feature names and their meanings.**

**Response**: We have created a comprehensive table summarizing all feature names and their clinical meanings. This table has been added to the Supplementary Materials (Table S4).

Table S4. Feature name and clinical meaning

| **Feature name** | **Clinical meaning** |
| --- | --- |
| Male Sex | Biological sex (Male) |
| Age | The standard count of time since birth. |
| AF burden | Atrial Fibrillation Burden. The percentage of time the heart spends in Atrial Fibrillation (AF) out of the total recording time. Reflects the severity of AF and atrial functional impairment. |
| Diabetes mellitus | A chronic metabolic disorder characterized by high blood glucose levels. Increases the risk of cardiovascular disease, kidney failure, and infections. |
| Congestive heart failure | A state where the heart cannot pump blood effectively. Often used to denote an acute exacerbation or severe cardiac function deterioration. |
| Chronic liver disease | Long-standing liver dysfunction affecting metabolism, coagulation, immune regulation, and detoxification. |
| Chronic kidney disease | Progressive decline in kidney function over more than three months. Affects fluid balance, electrolytes, and toxin clearance. |
| Chronic pulmonary disease | Long-term conditions (e.g., Chronic Obstructive Pulmonary Disease) that cause progressive reduction in respiratory function. Limits lung capacity and increases susceptibility to infection. |
| Cerebrovascular disease | Disorders of cerebral blood vessels that can cause ischemic or hemorrhagic brain injury and long-term neurologic deficits. (e.g., Stroke, Transient Ischemic Attack). |
| Cancer | Malignant disease associated with systemic inflammation, immune dysregulation, and increased risk of organ failure |
| Connective tissue disease | Autoimmune diseases (e.g., Lupus, Rheumatoid Arthritis) that cause systemic inflammation. |
| Dementia | Chronic, progressive decline in cognitive function. |
| Emergency department | Admission via the emergency department. |
| SOFA score | **Sequential Organ Failure Assessment score.** A scoring system that assesses the severity of acute organ dysfunction across six organ systems: Respiratory, Coagulation, Liver, Cardiovascular, Central Nervous System, and Renal. |
| Renal replacement therapy | **A set of medical treatments that replace the normal blood-filtering function of the kidneys** when they can no longer do so adequately |
| Ventilator use | Indicator of whether the patient required mechanical ventilation, reflecting the presence of severe respiratory failure or critical illness |
| Sepsis | Life-threatening organ dysfunction caused by a dysregulated host response to infection. |

2. **There are already many published models on in-hospital mortality; please explain how this study is different or novel compared to prior work. The top predictors mirror what has been repeatedly shown in published works (e.g., sepsis, age, SOFA). This limits the innovative value of your model.**

**Response**: Thank you for raising this important point regarding the novelty of our approach. We fully agree that numerous prediction models for in-hospital mortality have already been published, and many have repeatedly identified traditional predictors such as age, sepsis, and SOFA score. However, our work differs in several fundamental and innovative aspects that extend beyond prior studies.

Although numerous prediction models for in-hospital mortality have been proposed, most rely exclusively on conventional clinical variables such as age, sepsis, and SOFA score. These parameters are typically assessed intermittently and do not fully capture rapid physiological changes at the bedside. In contrast, our study leverages a continuously quantified AF burden derived from deep learning analysis of real-time ICU ECG monitoring. This approach reflects dynamic cardiac instability that may precede clinical deterioration, addressing a key limitation of static prognostic indices like the SOFA score, which depend on laboratory evaluations that cannot be updated in real time.

While continuous ECG monitoring is universally available in ICUs, its prognostic potential has not been fully utilized. AF is common in patients with critical illnesses and is well-documented to be associated with adverse outcomes; however, prior studies have predominantly treated AF as a binary event rather than a time-dependent exposure. Quantifying AF burden as a continuous variable enables a more detailed and clinically meaningful characterization of arrhythmia severity and duration. This is biologically plausible, as sustained AF can impair hemodynamics by eliminating effective atrial contraction, reducing ventricular filling, and accelerating ventricular rates, which collectively decrease cardiac output and lead to hemodynamic instability [1]. Therefore, AF burden represents an extension of a concept already familiar to clinicians, allowing its readily integration into existing bedside monitoring and decision-making processes.

Importantly, AF burden remained a substantial and independent predictor of mortality after adjustment for illness severity and well-established risk factors, highlighting its incremental prognostic value beyond traditional variables. In addition, external validation in an independent ICU cohort further supports the generalizability and clinical relevance of AF burden as a novel and actionable physiologic biomarker for mortality risk stratification in patients with critical illnesses.

To address the comment, we have incorporated additional clarification in the Discussion section, specifically at line 296 of the revised manuscript.

3. **Discuss the clinical significance of the key features highlighted in the SHAP plots; what do these findings imply in a medical context? SHAP and mortality common topics, find some new interpretability methods to interpret the model.**

**Response** Thank you for this valuable comment. Below we provide a clarification regarding the clinical significance of the SHAP findings and the role of AF burden. In the SHAP analysis, higher SOFA scores, mechanical ventilation use, and the presence of sepsis contributed positively to the predicted risk of in-hospital mortality, which is consistent with their roles as markers of multi-organ failure and critical illness severity. Notably, increasing AF burden was also associated with higher predicted mortality, suggesting that sustained or frequent AF episodes may reflect hemodynamic instability and impaired cardiovascular reserve in patients with critical illnesses. In the SHAP analysis, higher SOFA scores, mechanical ventilation, and sepsis showed strong positive contributions to predicted mortality, which is consistent with their established roles as indicators of illness severity and multi-organ dysfunction. Importantly, most of these features represent static admission-time variables, whereas AF burden uniquely functioned as a dynamic, real-time marker. An increasing AF burden consistently contributed to higher mortality risk, suggesting that frequent or sustained AF episodes reflect ongoing hemodynamic instability and worsening physiological status during the ICU stay. This finding highlights that real-time monitoring variables such as AF burden can complement conventional static predictors by capturing continuous clinical deterioration, thereby enhancing the model’s ability to identify high-risk patients more accurately. These results emphasize AF burden as both a clinically meaningful complication and a dynamic indicator of worsening mortality risk.

Regarding the reviewer’s suggestion to incorporate additional interpretability methods beyond SHAP, we fully agree that techniques such as integrated gradients, DeepLIFT, or counterfactual explanation frameworks may provide complementary perspectives on how predictive features—particularly dynamic physiologic measurements—contribute to risk estimation. While the present study focused solely on SHAP due to its established utility and strong applicability to tabular clinical data, we acknowledge the value of these alternative approaches and plan to explore them in future work to further strengthen interpretability.

In line with the reviewer’s comment, we have expanded the Discussion section (line 310) to provide additional context on the SHAP-based interpretability of our model.

4. **The model architectures are rather basic; consider adding some methodological novelty or advanced comparison.**

**Response**: Thank you for this constructive and insightful comment. Our primary modeling approach utilized tree-based ensemble methods and logistic regression because they provide high interpretability for clinical application and robustly address our primary research objective: investigating the association between AF burden and in-hospital mortality using tabular data. Crucially, the calculation of our core feature, the AF burden, already involved an advanced method: a SE-ResNet (Residual Network) architecture applied to the raw ECG signals. While we employed a standard SE-ResNet without extensive modification or comparison, this step demonstrates the incorporation of deep learning for robust feature engineering.

Furthermore, we would like to emphasize that the our study introduces and operationalizes **AF burden as a dynamic, continuously quantified marker**, which is a key conceptual novelty of our work. We clarified and highlighted this point in the discussion section to better convey its significance within critical care prognostication. In addition, we have added a concluding statement reflecting this novelty in line 293 of the revised manuscript.

Exploring more sophisticated or specifically tuned deep learning architectures, such as temporal models (e.g., LSTMs or Transformers) or customized CNN variants for enhanced AF detection and burden quantification, is a highly valuable endeavor and will be the focus of our future work.

5. **The image quality in the figures needs improvement (resolution).**

**Response**: All figures have been replaced with high-resolution versions to ensure clear visualization and improved readability.

6. **Indicate whether you have validated the model using an external dataset.**

**Response**: We have revised the manuscript to clearly emphasize that the model was validated using an external dataset, and we highlighted this information in both the Methods and Results sections for clarity. (line 411)

7. **Add statistical tables or figures (bar plots) summarizing the descriptive statistics of the input features, mainly the top contributing features.**

**Response**: In response to the reviewer’s suggestion, we have added feature importance plots for the top contributing variables to the Supplementary Materials (Figure S3). In addition, the descriptive statistics of the input features are already provided in **Table 1 as baseline characteristics,** which offer a comprehensive summary of the clinical features of the study cohort. Together, these additions enhance the clarity and interpretability of the model’s input features.

**Figure S3. Feature Importance (A) MIMIC-III dataset, (B) Yongin Severance Hospital**

(A)

(B)

**Reviewer’s Comment #1**

1. **In the Introduction and abstract the authors need, specify AF burden as a dynamic marker produced through real-time monitoring. In the introduction, clearly state the uniqueness of applying deep learning models to continuous and automatic computation of AF burden in critically ill patients compared to those utilizing intermittent monitoring devices.**

**Response**: Thank you for this helpful suggestion. In response, we have added the requested content to the Introduction section.

(line 75) Importantly, AF burden represents a dynamic marker that can only be accurately captured through continuous, real-time monitoring rather than intermittent ECG assessments. Prior studies relying on spot recordings or manual review were unable to reflect temporal fluctuations in AF episodes.

(line 82) Leveraging these advances, deep learning models now enable continuous and automatic computation of AF burden from full-length ICU ECG streams, offering a unique approach compared with conventional intermittent monitoring methods. However, this capability has not been explored in critically ill populations.

2. **The use consistent notation between the two data sets. For instance, sometimes MIMIC-III is also referred to as "training dataset" and Yongin Severance Hospital as "external validation dataset", which is right, but occasional use of "training dataset" without declaring MIMIC-III (e.g., Line 880) should be removed for clarity.**

**Response**: Thank you for the comment. We have reviewed and corrected all indicated parts of the manuscript.

3. **The Data Availability statement indicates that it is not feasible to share the external validation dataset openly due to the Personal Information Protection Act, understandable. However, in simple words, proclaim MIMIC-III as the primary, public dataset and categorically commit that the code used for the analysis (e.g., deployment of the deep learning model) will be shared to ascertain full reproducibility. (The mention of SQL code on GitHub is a solid start, yet the code of the ML/DL model is critical).**

**Response**: Thank you for the comment. We have made the model code publicly available, and it can now be accessed at the following repository:
<https://github.com/CMI-Laboratory/AF_burden>

4. **The discussion acknowledges that the study has a limitation in evaluating if AF burden-directed interventions reduce outcomes. However, more reinforcing the potential mechanism of action (e.g., hemodynamic instability, inflammatory response) linking high AF burden to mortality would strengthen the argument. While the ≥7.0% threshold for "high AF burden" is borrowed from an earlier study, its clinical and biological logic in the setting of this manuscript may be clarified further in the introduction or discussion.**

**Response:** We appreciate the reviewer’s insightful suggestion. We agree that further reinforcing the potential mechanisms linking AF burden to mortality strengthens the clinical interpretation of our findings. New-onset AF in patients with critical illnesses has been well documented to be associated with worse outcomes. A biologically plausible explanation is that AF burden may reflect underlying illness severity and systemic stress as a surrogate marker, given its frequent occurrence in the presence of elevated inflammatory responses, vasopressor use, electrolyte disturbances, and advanced organ dysfunction [1]. Additionally, AF burden may contribute to clinical deterioration, as sustained AF can eliminate effective atrial contraction, reduce diastolic filling, and increase ventricular rates, which together can impair cardiac output and worsen hemodynamic stability in vulnerable ICU patients [1]. These mechanisms support the clinical plausibility of the observed association between higher AF burden and increased mortality.

Regarding the ≥7% threshold, we want to clarify that our primary goal was not to propose a fixed cutoff for “high AF burden” but rather to characterize AF burden as a continuous physiologic exposure. To more clearly illustrate this relationship, and in response to the reviewer’s suggestion, we additionally performed a restricted cubic spline analysis during the revision [2]. This analysis demonstrated a progressive increase in mortality risk with higher AF burden, while also revealing significant nonlinearity. These findings indicate that AF burden does not exert a uniform risk effect across its range and suggest that a simple dichotomization may oversimplify its prognostic implications. This nonlinear pattern supports the use of flexible approaches, including our deep learning–based mortality prediction model, which can account for complex, non-linear relationships beyond the capability of linear models. We have revised the Discussion accordingly and included the spline results to visually highlight this continuous risk pattern.

**Figure S2. Restricted cubic spline showing the relationship between AF burden and in-hospital mortality in the MIMIC-III dataset**

Data were fitted using a logistic regression model with four knots placed at the 5th, 35th, 65th, and 95th percentiles of AF burden (reference = 5th percentile). Covariates that remained significant in the multivariable logistic regression model (Table 3) were included in the model to adjust for confounding.

CI, confidence interval; AF, atrial fibrillation.

5. **It is suggested that the authors need to mention about the technology foundation in the Introduction and Discussion, include a citation on the use of deep learning or AI in healthcare or IoT because it deserves the largest methodology. The following citation from the provided document is extremely relevant to the intersection of deep learning and health monitoring. The suggested reference paper from: “Deep Learning-Enabled Fetal Health Classification Through Sensor-Fused IoT. Mobile Radio Communications and 5G Networks”. This paper suggested explains where the lack of adequate research in AF burden and its clinical importance is expressed, to move to the solution using deep learning to the discussion to reinforce the present arguments in deep learning in healthcare and application of AI in risk stratification.**

**Response**: We appreciate the reviewer’s insightful suggestion. In response, we have expanded both the Introduction and Discussion to clarify the technological foundation of our work. Specifically, we added text describing how recent advances in deep learning for physiologic waveform analysis and IoT-enabled health-monitoring systems have enabled high-resolution interpretation of continuous bio signals. We have also incorporated the suggested reference, “Deep Learning-Enabled Fetal Health Classification Through Sensor-Fused IoT”, as it provides relevant context regarding the application of AI in continuous monitoring and its importance in addressing gaps in prior AF burden research. These revisions reinforce the methodological rationale for applying deep learning to ICU ECG telemetry and emphasize the contribution of AI-based approaches to modern risk stratification.

6. **Although deep learning forms the crux of the method, the exposition is primarily based on the existing deep learning architecture (ResNet/SE-ResNet). A brief clarification regarding how this computerized high-frequency load calculation is specifically bridging the limitation of traditional manual or intermittent monitoring in a busy ICU scenario would be beneficial.**

**Response**: Thank you for this valuable comment. The requested content has been incorporated into the Discussion section at line 339 of the revised manuscript.

(line 339) Importantly, this deep learning–based approach directly overcomes a key limitation of traditional AF assessment in the ICU, which often relies on intermittent rhythm checks or manual review of telemetry strips. Such methods cannot capture the rapid, high-frequency fluctuations in cardiac rhythm that occur during critical illness. By automatically analyzing every 10-second ECG segment, our model provides continuous, high-resolution quantification of AF burden that would be impractical to obtain manually, thereby reducing sampling bias and clinician workload in a busy ICU environment.

7. **Enhance Rationale for AF Burden Threshold: Provide a superior, evidence-based rationale in the discussion for employing the ≥7.0% threshold for high AF burden by explaining its clinical relevance or statistical derivation from prior work.**

**Response:** We agree that additional clarification regarding the ≥7.0% threshold would improve the interpretability of our findings. The ≥7% threshold was adopted from a prior multicenter study in which this value represented the median AF burden of the cohort. In that study, the authors used ≥7% as an operational cutoff for binary comparisons. Their findings showed that AF burden was a strong independent predictor of subsequent AF diagnosis and that the risk increased progressively with higher burden, supporting the interpretation of AF burden as a continuous risk factor.

Similarly, the aim of our study was not to define a fixed clinical cutoff for AF burden but to evaluate its continuous association with mortality risk. As shown in Fig S1, mortality increased steadily with higher AF burden, illustrating that AF burden behaves as a continuous risk factor. To further support and clarify this relationship, we performed a restricted cubic spline analysis. The results demonstrated a progressive increase in mortality risk with rising AF burden, albeit with significant nonlinearity.

Taken together, these findings reinforce that AF burden should be interpreted along its continuum, and the ≥7% threshold in our study served only as a supportive analytic reference point rather than a definitive clinical cutoff. We have revised the Discussion accordingly to more clearly reflect this rationale and to incorporate a more detailed appraisal of the prior study.

**Reviewer’s comment #2**

Methodological Transparency:

1. **Please clarify how AF burden was computed and justify the chosen cutoff (≥7.0%) for defining “high burden.” Was this threshold determined empirically, or derived from prior literature or ROC analysis?**

**Response**: The AF burden was calculated as the proportion of time spent in atrial fibrillation during continuous ECG monitoring, based on segment-level classifications generated by our SE-ResNet model. The ≥7% threshold was adopted from prior literature, where it served as an operational reference rather than a clinically validated cutoff. We would like to clarify that **our study was not intended to propose or define a specific clinical cutoff;** instead, our aim was to evaluate AF burden as a continuous exposure in relation to mortality risk. This has been more clearly stated in the revised manuscript.

2. **Provide more details on the deep learning model architecture (e.g., SE-ResNet-34), input parameters, and validation metrics (accuracy, sensitivity, specificity) used to ensure reliable AF detection.**

**Response**: Thank you for the comment. We have provided the additional information in the Methods section at line 437 of the revised manuscript.

(line 437) The SE-ResNet-34 model [27, 28] demonstrated the best overall performance (accuracy: 0.943, sensitivity: 0.937, specificity: 0.945, AUROC: 0.983) and was therefore selected as the final AF detection model for computing AF burden from ICU ECG waveforms (S2 Table). The SE-ResNet-34 architecture consisted of an initial 1-D convolutional layer (kernel size 7, 64 channels, stride 2), batch normalization, ReLU activation, and a max-pooling layer (kernel size 3, stride 2), followed by four residual stages with 3, 4, 6, and 3 blocks and channel widths of 64, 128, 256, and 512, respectively. In each residual block, two 1-D convolutional layers (kernel size 3) with batch normalization and ReLU were followed by a squeeze-and-excitation (SE) block, which applied global average pooling and two fully connected layers to adaptively reweight channel-wise features. A global average pooling layer over the temporal dimension and a final fully connected layer produced the binary AF prediction.

3. **Explain how ECG artifacts were managed and why patients with AF burden >0.9 were excluded.**

**Response**: Segments with poor signal quality or pacemaker rhythms were excluded from the analysis. The signal quality was assessed using a high-pass filter to remove the baseline wander, setting the cutoff frequency at 0.5 Hz. A power line filter was employed to eliminate the 50-Hz power line noise. Z-normalization was performed using the mean and standard deviation. Cases in which the AF burden could not be calculated due to incomplete ECG data were excluded. Furthermore, patients with an AF burden > 0.9 were also excluded. An AF burden above this level is highly suggestive of persistent AF, which is defined as AF lasting longer than 7 days and not self-terminating [3]. In such cases, AF is continuously present regardless of short-term changes in clinical condition, making it less appropriate to use AF burden as a dynamic predictor of outcomes. Given the lack of formal historical diagnostic information (e.g., past ECG data and the physician's diagnosis) to reliably exclude all persistent AF cases, the > 0.9 threshold was employed based on this clinical rationale.

Confounding and Adjustment:

4. **Expand on the rationale for including variables such as SOFA score, sepsis, and ventilator use in the multivariable model. Discuss potential multicollinearity, and report VIFs if available.**

**Response:** In our multivariable analysis, we included the SOFA score as an index reflecting disease severity in patients with critical illnesses. Because the SOFA score is derived from six organ-specific components—three of which (respiratory, renal, and cardiovascular dysfunction) incorporate information related to ventilator use, renal replacement therapy (RRT), and vasopressor use—these variables are inherently correlated with SOFA. For this reason, ventilator use, RRT, and vasopressor therapy were not included in the original model to avoid redundancy and potential multicollinearity.

We agree that additional multicollinearity may exist between SOFA score, sepsis, and ventilator use, as the presence of sepsis often influences multiple organ dysfunction domains included in the SOFA calculation. In response to the reviewer’s suggestion, we assessed potential multicollinearity across all included variables by calculating variance inflation factors (VIFs). All VIF values were <5, indicating no meaningful multicollinearity requiring correction. We have added these VIF results to the footnote of Table 3.

5. **Consider adjusting for additional confounders such as vasoactive drug use, baseline cardiac history, or renal replacement therapy.**

**Response:** As noted in our response to Comment 4, we initially included the SOFA score in the multivariable model as a comprehensive indicator of disease severity. Because ventilator use, RRT, and vasopressor therapy are already incorporated within the respiratory, renal, and cardiovascular components of the SOFA score, including these variables simultaneously may lead to redundancy and potential multicollinearity. Therefore, these variables were excluded from the original model.

We agree, however, that variables such as vasopressor use or RRT may have prognostic relevance beyond what is captured by the SOFA score. Incorporating such clinically meaningful variables—even when conceptually overlapping with SOFA—is a common analytic strategy for evaluating their incremental contribution to patient outcomes. In line with the reviewer’s suggestion, we constructed an expanded model that included these additional variables. We fully agree with the reviewer that baseline cardiac history is an important prognostic factor and is conceptually relevant when evaluating outcomes related to AF and mortality. Unfortunately, this information was not available in our dataset, and therefore could not be included into the multivariable analysis. We have now acknowledged this as a limitation in the revised manuscript.

We evaluated multicollinearity using variance inflation factors (VIFs), and all variables demonstrated VIF values <10, indicating no meaningful multicollinearity. Importantly, the main finding remained consistent: high AF burden continued to be significantly associated with increased mortality in the expanded model. We have updated the Results accordingly to reflect these additional analyses.

**Revised Table 3. Risk factors for in-hospital mortality in patients with critical illness**

|  | **Univariable analysis** | | **Multivariable analysis** | |
| --- | --- | --- | --- | --- |
|  | **OR (95% CI)** | ***P*-value** | **aOR (95% CI)** | ***P*-value** |
| Age | 1.03 (1.03–1.04) | <0.001 | 1.03 (1.03–1.04) | <0.001 |
| Male sex | 0.92 (0.80–1.06) | 0.241 | 0.90 (0.77–1.06) | 0.220 |
| MICU | 1.75 (1.51–2.02) | <0.001 | 1.10 (0.92–1.31) | 0.305 |
| SICU | 0.96 (0.81–1.13) | 0.595 |  |  |
| CCU | 0.96 (0.80–1.14) | 0.637 |  |  |
| CSRU | 0.35 (0.26–0.45) | <0.001 | 0.23 (0.17–0.32) | <0.001 |
| TICU | 1.02 (0.79–1.30) | 0.865 |  |  |
| Admission via ED | 4.23 (3.09–5.96) | <0.001 | 2.54 (1.79–3.71) | <0.001 |
| Charlson Comorbidity Index | 1.18 (1.15–1.21) | <0.001 | 1.09 (1.06–1.13) | <0.001 |
| High AF burden | 2.35 (2.03–2.72) | <0.001 | 1.63 (1.36–1.95) | <0.001 |
| SOFA score | 1.30 (1.27–1.33) | <0.001 | 1.21 (1.18–1.25) | <0.001 |
| Ventilator use | 4.20 (3.55–4.99) | <0.001 | 3.90 (3.20–4.76) | <0.001 |
| Renal replacement therapy | 2.39 (1.84–3.08) | <0.001 | 0.92 (0.67–1.27) | 0.629 |
| Vasopressor use | 3.85 (3.30–4.49) | <0.001 | 1.06 (0.86–1.32) | 0.575 |
| Sepsis | 3.71 (3.21–4.30) | <0.001 | 1.36 (1.14–1.62) | <0.001 |

All variance inflation factors were <5, and the Hosmer–Lemeshow test indicated no significant lack of fit (*P* = 0.203).

Model Interpretability and Generalizability:

6. **Provide quantitative data on feature importance (not only SHAP plots) to highlight the predictive contribution of AF burden relative to other variables.**

**Response**: Thank you for the valuable feedback. As the editor provided similar comments, we have added the feature importance results for both the MIMIC-III and the Yongin Severance cohorts. These have been included in the revised Supplementary Figure S3.

7. **In the external validation cohort, please describe ECG data collection parameters and confirm that preprocessing and classification pipelines were identical to the MIMIC-III cohort.**

**Response**: We appreciate the reviewer’s insightful comment. The corresponding content has been incorporated into the Methods section at line 463 of the revised manuscript.

(line 463) For the external validation cohort, the identical preprocessing pipeline and AF classification model used for the MIMIC-III cohort were applied without any modification, including the same filtering procedures, segmentation strategy, normalization method, and the pretrained SE-ResNet-34 classifier. AF burden in the external cohort was computed using the same algorithm, ensuring methodological consistency across cohorts.

Clinical Implications:

8. **The discussion could be strengthened by explaining how real-time AF burden monitoring might be integrated into ICU alert systems or decision support tools.**

**Response**: Thank you for the helpful comment. The requested content has been added at line 311 of the revised manuscript.

(line 311)	AF burden also emerged as an important contributor, functioning as a dynamic real-time marker rather than a static admission-time variable. Increasing AF burden was associated with a steadily higher predicted mortality risk, indicating that sustained or frequent AF episodes capture ongoing hemodynamic instability during the ICU stay. These results further support the added value of continuous monitoring–derived variables in mortality prediction models.

**Reference**

**1**.	Bosch NA, Cimini J, Walkey AJ. Atrial Fibrillation in the ICU. Chest. 2018;154(6):1424-34. <https://doi.org/10.1016/j.chest.2018.03.040> PMID:29627355

**2**.	Gauthier J, Wu QV, Gooley TA. Cubic splines to model relationships between continuous variables and outcomes: a guide for clinicians. Bone Marrow Transplant. 2020;55(4):675-80. <https://doi.org/10.1038/s41409-019-0679-x> PMID:31576022

**3**.	Van Gelder IC, Rienstra M, Bunting KV, Casado-Arroyo R, Caso V, Crijns H, et al. 2024 ESC Guidelines for the management of atrial fibrillation developed in collaboration with the European Association for Cardio-Thoracic Surgery (EACTS). Eur Heart J. 2024;45(36):3314-414. <https://doi.org/10.1093/eurheartj/ehae176> PMID:39210723
